# Population Pharmacokinetics and Model-Informed Precision Dosing of Clobazam Based on the Developmental and Genetic Characteristics of Children with Epilepsy

**DOI:** 10.3390/pharmaceutics17070813

**Published:** 2025-06-23

**Authors:** Yali Tuo, Xiaolong Yu, Sichan Li, Jun Wang, Maochang Liu, Xinwen Song, Jiehui Ma, Yang Wang, Zhisheng Liu, Dan Sun

**Affiliations:** 1 Department of Pharmacy, Wuhan Children’s Hospital, Tongji Medical College, Huazhong University of Science and Technology, Wuhan 430016, China; tuoyali@zgwhfe.com (Y.T.); hunter234@126.com (S.L.); wangjun2@zgwhfe.com (J.W.); liumaochang@zgwhfe.com (M.L.); songxinwen@zgwhfe.com (X.S.); 2 Emergency and Critical Care Medical Center, Wuhan Children’s Hospital, Tongji Medical College, Huazhong University of Science and Technology, Wuhan 430016, China; yuxiaolong@zgwhfe.com; 3 Department of Neurology, Wuhan Children’s Hospital, Tongji Medical College, Huazhong University of Science and Technology, Wuhan 430016, China; majiehui@zgwhfe.com; 4 Drug Clinical Trial Institution, Wuhan Children’s Hospital, Tongji Medical College, Huazhong University of Science and Technology, Wuhan 430016, China; wangyang@zgwhfe.com

**Keywords:** clobazam, population pharmacokinetics, CYP2C19 genotype, body weight, dosing optimization, children

## Abstract

**Background/Objectives:** This study aimed to characterize the pharmacokinetic profiles of clobazam (CLB) and its active metabolite, N-desmethylclobazam (N-CLB), by establishing a population pharmacokinetic (PPK) model in Chinese children with epilepsy to propose individualized dosing regimens that achieve better clinical outcomes. **Methods**: This study examined plasma samples collected from 103 pediatric patients with refractory epilepsy undergoing CLB treatment. The plasma concentrations of CLB and its active metabolite N-CLB were measured. The developmental characteristics, CYP2C19 genotype, concomitant medications, and liver and kidney function indicators of the children with epilepsy were considered potential factors affecting the pharmacokinetic characteristics of CLB and N-CLB and analyzed using a PPK modeling approach. **Results**: A total of 156 samples were attained for PPK model development. The pharmacokinetic profiles of CLB and N-CLB were described using a tandem one-compartment model with first-order elimination. Body weight and CYP2C19 genotype showed statistical significance for CLB and/or N-CLB clearance. The N-CLB/CLB metabolic ratios of AUC_24h_, C_min_, and C_max_ in a steady state were as follows: normal metabolizers (NMs) < intermediated metabolizers (IMs) < poor metabolizers (PMs). The final model achieved good prediction performance and stability. Monte Carlo simulations demonstrated that the trough concentrations of CLB and N-CLB in children with epilepsy could reach satisfactory target values under varying dose regimens in CYP2C19 NMs and IMs, whereas there was a failure to achieve the desired trough concentrations of CLB and N-CLB simultaneously in CYP2C19 PMs due to the accumulation of N-CLB. **Conclusions**: Body weight and CYP2C19 genotype had an impact on CLB and/or N-CLB clearance in children with epilepsy. To ensure safe treatment, it is recommended to use the concentration of N-CLB as the target indicator for therapeutic drug monitoring and dose adjustments in CYP2C19 PMs. These results provide evidence for guiding the precise use of CLB.

## 1. Introduction

Clobazam (CLB), a 1,5-benzodiazepine, has been widely prescribed for the treatment of Lennox–Gastaut syndrome, Dravet syndrome, epilepsy with myoclonic–astatic seizures (EMAS), and other refractory epilepsies for several years in foreign countries [1]. CLB is usually combined with a wide range of antiepileptic drugs, such as valproic acid, lamotrigine, and oxcarbazepine. Compared with 1,4-benzodiazepines, such as diazepam and clonazepam, CLB shows an improved side effect profile and is associated with a lower incidence of nervous and skin system complications, such as Stevens–Johnson syndrome (SJS) or toxic epidermal necrolysis (TEN); sedation and physical discomfort; and physical dependence [2,3,4]. Given its potent antiepileptic properties with fewer benzodiazepine-related side effects, CLB was approved in China for use as adjunctive therapy for the treatment of refractory epilepsies.

CLB is rapidly and extensively absorbed after oral administration. The maximum serum concentration of CLB is achieved within 0.5–4 h [5,6], and the maximum serum concentration of its main active metabolite, N-desmethylclobazam (N-CLB), is achieved between 3 and 56 h [5,7]. CLB distributes extensively in body tissues, and the degree of protein binding for CLB and N-CLB is 80–90% and 70%, respectively [8]. CLB and its metabolites are excreted in urine (major pathway) and feces (minor pathway) [9]. Only the unbound molecules of CLB and N-CLB can cross the blood–brain barrier [8]. Both CLB and N-CLB exert their effects by binding to GABA_A_ receptors directly, potentiating inhibitory neurotransmission [10]. Although the serum concentration of N-CLB is 3 to 5 times higher than that of CLB, N-CLB has less pharmacological activity, at one-fifth of the activity of CLB. The half-life of N-CLB (71 to 82 h) is approximately twice that of CLB (36 to 42 h) in human bodies [11]. In multiple-dose studies, CLB reaches steady-state concentrations within one week of treatment initiation, while N-CLB may take up to three weeks to reach steady-state concentrations [11,12].

In humans, CLB is primarily metabolized by CYP3A4, with the partial involvement of CYP2C19, to produce the active metabolite N-CLB [9,13]. In addition to CYP3A4 and CYP2C19, other single-nucleotide polymorphisms (SNPs) may also be associated with variations in CLB/N-CLB plasma concentration. For example, ABCB1 polymorphisms influence responsiveness to antiepileptic drugs [14]. P-gp (MDR1), which is mediated by ABCB1, is upregulated in excretory organs, indicating its key function in drug clearance and its potential association with the plasma level of antiseizure medications [15]. These genetic factors make CLB prone to pharmacogenetic variability. N-CLB is further hydroxylated to inactive 4′-hydroxydesmethylclobazam, predominantly by CYP2C19 [9,13]. CYP2C19 is polymorphically expressed, and, according to a previous report [16], individuals can be classified into five subgroups based on their ability to metabolize it: ultrarapid metabolizers (UMs: *17/*17), rapid metabolizers (RMs: *1/*17), normal metabolizers (NMs: *1/*1), intermediate metabolizers (IMs: *1/*2, *1/*3, *2/*17, or *3/*17), and poor metabolizers (PMs: *2/*2, *2/*3, or *3/*3). It has been reported that the plasma levels of N-CLB in PMs are five times higher compared to individuals with NMs, increasing the risk of adverse reactions [16]. Given the significantly higher prevalence of PMs in Asian people (about 15% to 25%) compared to White people (2% to 5%), it is important to determine CYP2C19 genotypes, particularly in Asian populations, to minimize potential adverse effects [16].

It is beneficial to maintain appropriate levels of exposure to CLB and N-CLB in vivo to ensure the seizure freedom rate and safety of antiepileptic treatment. Generally, steady-state trough concentrations are required to reach 30–300 μg·L^−1^ for CLB and 300–3000 μg·L^−1^ for N-CLB [12,17]. The use of precision dosing guided by population pharmacokinetic models is a common approach in pediatrics to increase the probability of reaching therapeutic concentrations of antiepileptic drugs. Compared with traditional pharmacokinetics, population pharmacokinetics (PPKs) can quantify intrinsic and extrinsic factors affecting pharmacokinetics by utilizing sparse data [18]. Several PPK models for CLB in French, Japanese, and American populations have been developed, with a focus on CYP3A4 and CYP2C19 [19,20,21]. No studies have included Chinese patients, and there are few PPK models for CLB associated with children [19]. This restricts the implementation of individualized CLB dosing in the Chinese pediatric population because of a lack of understanding of the pharmacokinetic characteristics of CLB in this population.

This study was the first application of CLB in Chinese pediatric patients with epilepsy based on real-world data. It comprehensively analyzed the impact of children’s development and genetic characteristics on the pharmacokinetic characteristics of CLB and N-CLB in vivo through PPK modeling and further proposed optimized dosing regimens based on the model to improve the probability of achieving the target exposure level.

## 2. Materials and Methods

### 2.1. Study Population

This study was a prospective, single-center study conducted at Wuhan Children’s Hospital, China, between December 2022 and March 2024. Pediatric epilepsy patients (aged ≤ 18 years old) who received CLB treatment were enrolled. These patients were diagnosed with Lennox–Gastaut syndrome, Dravet syndrome, infantile spasms, or other refractory epilepsies. The exclusion criteria were as follows: (1) patients who received treatments that might influence the pharmacokinetic process of CLB; (2) patients who were participating in other clinical studies during CLB treatment; (3) patients with poor medication adherence; and (4) patients for whom key research data were missing [22].

The study protocol complied with the Declaration of Helsinki and was approved by the Wuhan Children’s Hospital Ethics Committee (protocol number: 2021R142-E02/03). The guardians or relatives of all patients provided their written informed consent before enrollment in this study.

### 2.2. Dose and Samples

CLB tablets (Yichang Humanwell Pharmaceutical Co., Ltd., Yichang, China) were administered twice daily if the dose was greater than 5 mg. The starting dose was 5 mg for patients weighing ≤ 30 kg and 10 mg for those >30 kg. The dose was then individually adjusted according to efficacy and tolerability. This study was conducted using an opportunistic sampling approach. To monitor the plasma concentration of CLB, 2 mL samples derived from scavenged residual blood specimens for biochemical testing during the safety follow-up period were placed in EDTA-K2 tubes for pharmacokinetic analysis and genotype detection. The patients’ dosing histories and sampling times were accurately recorded. After centrifugation at 450× *g* × 10 min, plasma and blood cell sediment were separated and stored at −80 °C until analysis.

An electronic medical record system was used to acquire the patients’ information, including their demographic characteristics (age, gender, weight, and height), blood biochemical information (liver and kidney function), adherence to a ketogenic diet, and whether they were treated with concomitant antiepileptic drugs. The estimated glomerular filtration rate (eGFR) was calculated using the modified Schwartz formula [eGFR (mL/min·1.73 m^2^) = 0.413 × Height/Serum creatinine] [23].

### 2.3. Analysis of CLB and N-CLB Plasma Concentrations

Simultaneous measurements of the CLB and N-CLB concentrations in the human plasma samples were conducted using high-performance liquid chromatography–tandem mass spectrometry (HPLC-MS) at Caresunpharma Co., Ltd. (Wuhan, China). A brief description of the analytical method is provided as follows: A 50 μL volume of plasma was mixed with 50 μL of 50% methanol and then vortexed for at least 3 min. Next, 300 µL of acetonitrile was added, and the sample was vortexed for at least 10 min. After centrifugation at 1800× *g* × 10 min at 4 °C, 100 µL of the supernatant was collected, mixed with 150 µL of purified water, and shaken for at least 5 min prior to detection.

A Shimadzu HPLC system (Kyoto, Japan), consisting of binary LC-30AD pumps, an online DGU-20A degasser, a column oven, and a refrigerated (4 °C) auto-sampler system, was used. Chromatographic separation was performed on a Welch Xtimate-C18 column (2.1 × 50.0 mm, 3 μm) at a temperature of 40 °C, utilizing a mobile phase of deionized water supplemented with 5 mM ammonium bicarbonate (solvent A) and acetonitrile (solvent B). The gradient was conducted at a flow rate of 0.4 mL·min^−1^ as follows: 0.00–1.50 min 40–55% (B), 1.50–1.51 min 55–90% (B), 1.51–2.30 min 90% (B), 2.30–2.31 min 90–40% (B), and 2.31–3.50 min 40% (B). The injection volume was 5 μL.

Mass spectrometric detection was performed on a QTRAP 5500 mass spectrometer (AB Sciex, Framingham, MA, USA) equipped with an electrospray ionization (ESI) interface in positive ionization mode. Multiple-reaction monitoring (MRM) was used to monitor analytes and an internal standard. The mass conditions were as follows: ion spray voltage, 5500 V; ion source gas 1 (N_2_), 50 psi; ion source gas 2 (N_2_), 55 psi; source gas temperature, 500 °C; curtain gas (N_2_), 40 psi; collision gas (N_2_), medium; entrance potential, 10 V; and cell exit potential, 10 V. The selected mass transitions were *m*/*z* 301.2 → 259.1 for CLB, *m*/*z* 306.2 → 264.1 for CLB IS, *m*/*z* 287.2 → 245.1 for N-CLB, and *m*/*z* 292.2 → 250.1 for N-CLB IS. The declustering potential (DP) was set at 50 V. The collision energy (CE) was 45 V for CLB and IS and 28 V for N-CLB and IS. Analyst 1.6.3 software was used to control the instrument and acquire the data. The accuracy variation coefficient for determining both compounds was <5%. The quantitative ranges of this method were 3–1200 μg·L^−1^ for CLB and 40–16,000 μg·L^−1^ for N-CLB.

### 2.4. Genetic Analyses

Genomic DNA was extracted from the blood samples using an Ezup Column Blood Genomic DNA Purification kit (Sangon Biotech, Shanghai, China; Cat. No. B518253), following the manufacturer’s instructions. Polymorphisms were genotyped via Sanger sequencing using an ABI 3730xl DNA Analyzer (Applied Biosystems, Foster City, CA, USA). Several functional SNPs were selected, including rs1128503 (ABCB1 1236C > T), rs2032582 (ABCB1 2677G > T/A), rs1045642 (ABCB1 3435C > T), rs2740574 (CYP3A4*1B C > T), rs2242480 (CYP3A4*1G 20230T > C), rs4244285 (CYP2C19*2 681G > A), rs4986893 (CYP2C19*3 636G > A), rs12248560 (CYP2C19*17 −806C > T), rs2279020 (GABRA1 IVS11+15 A > G), rs279858 (GABRG1 T > C), rs11503014 (GABRA2 C > G), rs2229944 (GABRB2 G > A), rs211014 (GABRG2 C > A), and rs211037 (GABRG2 588C > T). For CYP2C19, the genotypes were classified into five groups: UMs (*17/*17), RMs (*1/*17), NMs (*1/*1), IMs (*1/*2, *1/*3, *2/*17, *3/*17), and PMs (*2/*2, *2/*3, *3/*3). Detailed information on the SNPs is shown in Supplementary Table S1.

### 2.5. PPK Model Establishment

PPK analysis was performed using Phoenix NLME software (v8.3.4, Certara USA, Inc., Princeton, NJ, USA). R software (version 3.5.1, https://www.r-project.org/ (accessed on 2 July 2018)) was used for data management and plotting.

This study attempted to characterize the pharmacokinetic profile of CLB in pediatric patients using one- or two-compartment structural models. Ultimately, a one-compartment model with first-order elimination was employed to describe the pharmacokinetics of CLB and N-CLB due to a lack of data on absorption and distribution phases. The absorption rate constant (Ka) was not estimated. The material balance equations are shown in Equations (1)–(3).

dAa/dt = −Ka × Aa × F(1)

dA_CLB_/dt = Ka × Aa × F − CL_CLB_ × A_CLB_/V_CLB_ (2)

dA_N-CLB_/dt = CL_CLB_ × A_CLB_ × F_m_/V_CLB_ − CL_N-CLB_ × A_N-CLB_/V_N-CLB_ (3)

where Aa represents the dose of CLB and Ka is the absorption rate constant. Since most of the pharmacokinetic observation values in this study were obtained in the elimination phase, Ka was fixed at 1.99, according to [19]. F is the oral bioavailability of CLB. A_CLB_ is the amount of CLB in the central compartment. CL_CLB_/F is the apparent clearance of CLB. V_CLB_/F is the apparent volume of distribution of CLB. A_N-CLB_ is the amount of N-CLB in the metabolic compartment. F_m_ is the dosage conversion fraction from CLB to N-CLB. Since the amount of CLB converted to N-CLB was not clear, F_m_ was not estimated in this study. CL_N-CLB_/F_m_ is the apparent clearance of N-CLB. V_N-CLB_/F_m_ is the apparent volume of distribution of N-CLB.

The interindividual variability was described exponentially as follows:

Pi = θ × exp(ηi)(4)

where Pi is the individual estimate of the PK parameter, θ is the typical population value, and ηi is the interindividual deviation, which is presumed to be normally distributed with a mean of 0 and variance of ω^2^.

The intraindividual variability was described with a proportional error model as follows:

Y = IPRED × (1 + ε)(5)

where Y denotes the observed values, IPRED is the individualized predicted values, and ε represents a randomly distributed variable with a mean of 0 and variance of σ^2^.

### 2.6. Analyses of Covariates

The likelihood ratio test was employed to screen the covariates in the stepwise method. In a forward inclusion process, a covariate was incorporated into the base model if the objective function value (OFV) decreased by more than 3.84 and was considered to have a significant effect (χ^2^ distribution, *p* < 0.05, *df* = 1). A full model was obtained by incorporating all selected covariates. Next, backward elimination was adopted to re-evaluate these covariates, in which a covariate was retained if the OFV increased by more than 6.63 (χ^2^ distribution, *p* < 0.01, *df* = 1). The final model was established using this stepwise approach.

In the process of model establishment, all variables previously described in the data collection, including demographics, clinical indicators, concomitant drugs, adherence to a ketogenic diet, and the nine SNPs, were investigated. Formulas (6) and (7) were used for the introduction of continuous variables and categorical variables into the model, respectively.

(6) $$\theta_{i}=\theta \;\times \;{\left(\frac{{Cov}_{j}}{{Cov}_{median}}\right)}^{\theta_{cov}}$$

θ_i_ = θ × exp(*θ_cov_*) (7)

where θ_i_ represents the predicted population pharmacokinetics value, θ represents the population typical value, Cov_j_ represents the value of the jth continuous covariate, Cov_median_ represents the median of the Cov_j_, and θ_cov_ represents the fixed effect of the covariate on the parameter.

### 2.7. Model Evaluation

The final model was evaluated through nonparametric bootstrap analysis, goodness-of-fit (GOF) plots, visual predictive checks (VPCs), and prediction-corrected VPCs (pc-VPCs). GOF plots were composed for the observed concentration versus the individual predicted concentration (IPRED), the observed concentration versus the population-predicted concentration (PRED), the conditional weighted residuals (CWRESs) versus the PRED, and the CWRESs versus the time after dose. The nonparametric bootstrap method, VPCs, and pc-VPCs were used to assess the stability and accuracy of the final model, and resampling from the original dataset was performed 1000 times. The medians and 95% confidence intervals (from 2.5% to 97.5% percentiles), estimated via the bootstrap method, were compared with the parameter estimates of the final PPK models in the bootstrap analysis.

### 2.8. Simulation and Dosing Optimization

To analyze the influence of genetic factors on the metabolism of CLB in vivo, the metabolic ratios of N-CLB to CLB regarding the area under the concentration–time curve (AUC) from 0 to 24 h (AUC_24h_), the maximum plasma concentration (C_max_), and the trough concentration (C_min_) in CYP2C19 NMs, IMs, and PMs were estimated using the maximum a posteriori Bayesian method. Monte Carlo methods were used to simulate optimal choices under different scenarios according to the covariates incorporated into the final model. In addition, the probability of target attainment (PTA) for CLB and N-CLB trough concentration values was calculated according to body weight and CYP2C19 genotype. The current target plasma concentration ranges are commonly defined as 30–300 μg·L^−1^ for CLB and 300–3000 μg·L^−1^ for N-CLB [12,17]. In the context of individual patient response to therapy, laboratory alert levels of 500 μg·L^−1^ for CLB and 5000 μg·L^−1^ for N-CLB were considered safety targets [19,24].

## 3. Results

### 3.1. Participants

The major demographic and clinical characteristics of the 103 patients in the present study are displayed in Table 1. Among these 58 males and 45 females, the mean age was 5.94 ± 3.15 years (range: 0.85–16.75 years), and the mean body weight was 22.94 ± 10.48 kg (range: 6.60–73.00 kg). In total, 302 concentrations were measured (154 for CLB and 148 for N-CLB). Specifically, 68 patients were sampled once, 21 were sampled twice, and 12 were sampled three times. The remaining two were sampled four and six times, respectively. The median (range) of the measured CLB concentrations was 137.01 μg·L^−1^ (15.40–495.00 μg·L^−1^), while the measured N-CLB concentrations ranged from 55.90 to 7060.00 μg·L^−1^, with a mean of 1611.56 μg·L^−1^. Among all the patients, 80.58% received concomitant valproic acid treatment and 28.16% received concomitant lamotrigine, whereas the remaining received perampanel (24.27%), levetiracetam (18.45%), topiramate (17.48%), and others (Supplementary Table S2). In total, 6.80% of patients adhered to a ketogenic diet. As many as 75.73% (78) of the patients used three or more antiepileptic drugs, indicating that the majority of the enrolled patients had refractory epilepsy.

Twelve SNPs involved in the absorption, metabolism, and clearance of CLB and N-CLB were analyzed. All the observed frequencies were consistent with the Hardy–Weinberg equilibrium (*p* > 0.05). A total of 103 patients provided genetic results, and the CYP2C19 genotype information is shown in Table 1. In total, 2, 41, 45, and 15 patients were CYP2C19 RMs, NMs, IMs, and PMs, respectively. Due to the limited sample size of RMs, the impact of this metabolic type on clearance was not included in the PPK analysis. SNP information of ABCB1, CYP3A4, and GABRA1, GABRG1, GABRB2, and GABRG2 was also detected and listed in Supplementary Table S1 for further PPK modeling.

### 3.2. PPK Model Development

As the pharmacokinetic data for CLB and N-CLB were predominantly collected during the elimination phase, a robust pharmacokinetic analysis of absorption and distribution characteristics could not be performed. The initial one-compartment model yielded OFV = 4206.28, AIC = 4222.28, and BIC = 4251.96. Comparative analysis with the two-compartment model (OFV = 4196.62, AIC = 4220.62, and BIC = 4265.14) revealed only marginal improvement (ΔOFV = −9.66, ΔAIC = −1.66, and ΔBIC = 13.18), with comparable information criteria values. Furthermore, the two-compartment model exhibited poor parameter precision (RSE% > 100%) due to sampling constraints. Thus, a one-compartment model for both CLB and N-CLB with first-order elimination for CLB was chosen for the basic infrastructure model. Demographic and genetic polymorphism data were assessed. Given the changes in growth and metabolism in pediatric patients, a simple exponential model was selected (Supplementary Table S3). For the base model, the OFV was 4206.28. Covariate analysis indicated that age, gender, body surface area, and hepatic and renal function had no significant impact on the pharmacokinetic parameters of CLB and N-CLB. The coadministration of several antiepileptic drugs, as well as SNPs of ABCB1, CYP3A4, and GABA receptors, also had no effect on the pharmacokinetics (Supplementary Figures S1 and S2). The addition of the concomitant medications and the above SNPs failed to reduce the model’s OFVs significantly below the threshold. The lowest OFV was 4119.90 when CYP2C19 was added to the model after stepwise analysis (Supplementary Table S3). Body weight had a significant influence on the V/F, CL/F, V/F_m_, and CL/F_m_, while the CYP2C19 genotype only significantly affected CL/F_m_. The estimates of the final model are listed in Table 2. The relative standard errors were below 40% for all parameters, indicating that they were well estimated. The absorption rate constant of CLB (Ka) was fixed at 1.99 h^−1^ based on the existing literature [19]. The volumes of distribution of CLB and its metabolite N-CLB were 92.07 L and 1.84 L, respectively. The clearance rates of CLB and its metabolite N-CLB were 5.66 L·h^−1^ and 1.01 L·h^−1^, respectively. The final model is as follows:

(8) $$K\alpha \left(1/h\right)=1.99$$

(9) $$V_{\mathrm{CLB}}/F(L)=92.07\times {\left(\frac{Weight}{70}\right)}^{1.0}$$

(10) $${\mathrm{CL}}_{\mathrm{CLB}}/F(L\cdot h^{-1})=5.66\times {\left(\frac{Weight}{70}\right)}^{0.75}$$

(11) $$V_{N-\mathrm{CLB}}/F_{m}(L)=1.84\times {\left(\frac{Weight}{70}\right)}^{1.0}$$

(12) $${\mathrm{CL}}_{N-\mathrm{CLB}}/F_{m}(L\cdot h^{-1})=1.01\times {\left(\frac{Weight}{70}\right)}^{0.75}\times \exp(\theta_{\mathrm{CYP2C19},\mathrm{genotype}})$$

where V_CLB_/F represents the apparent volume of distribution of CLB. CL_CLB_/F represents the apparent clearance of CLB. V_N-CLB_/F_m_ represents the apparent volume of distribution of N-CLB. CL_N-CLB_/F_m_ represents the apparent clearance of N-CLB.

### 3.3. Validation of Final PPK Model

**Figure 1:**
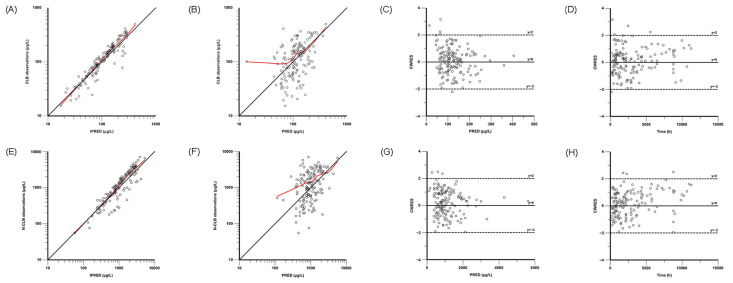
Goodness-of-fit plots of the final model for CLB (**upper** panel) and N-CLB (**lower** panel). (**A**,**E**) Observed concentration versus individual-predicted concentration (IPRED); (**B**,**F**) observed concentration versus population-predicted concentration (PRED); (**C**,**G**) conditional weighted residuals (CWRES) versus PRED; (**D**,**H**) CWRES versus time. The black straight line represents the unity line (y = x), and the red curve represents the LOESS line (Locally Weighted Regression Curve) in (**A**,**B**,**E**,**F**).

**Figure 2:**
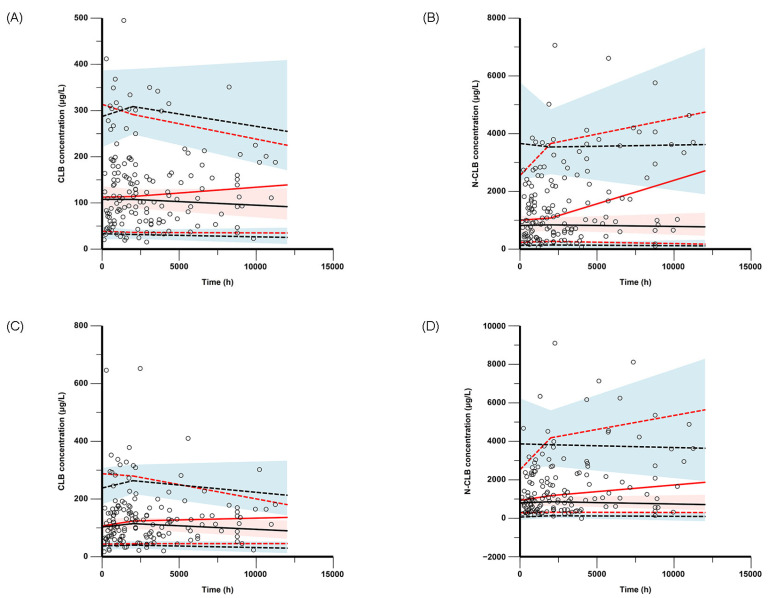
Visual predictive check (VPC) of serum concentrations versus time for (**A**) CLB and (**B**) N-CLB. Prediction-corrected VPC (pc-VPC) results for (**C**) CLB and (**D**) N-CLB. Observation data (circles); red dashed lines, 10th and 90th percentiles of observed concentrations; red solid lines, 50th percentile of observed concentrations; black dashed lines, 10th and 90th percentiles of simulated concentrations; black solid lines, 50th percentile of simulated concentrations; semitransparent field, 90% confidence intervals of the simulated 10th, 50th, and 90th percentiles from 1000 simulations.

The GOF plots show that the observed plasma concentrations closely matched the model-predicted concentrations, indicating that the model accurately represents the data. The model’s CWRES fell within ±2 standard deviations with a symmetrical distribution on both sides of y = 0, indicating a good fit to the original dataset (Figure 1). The model’s VPC and pc-VPC plots are presented in Figure 2, showing that the majority of the observed concentrations are distributed between the 10th and 90th percentiles of the simulated concentrations. Moreover, the estimated parameter values from the final model fall within the 95% confidence interval of the nonparametric bootstrap and are closely concordant with the median (Table 2). These results suggest that the model satisfactorily described the pharmacokinetics of CLB and N-CLB.

**Figure 3:**
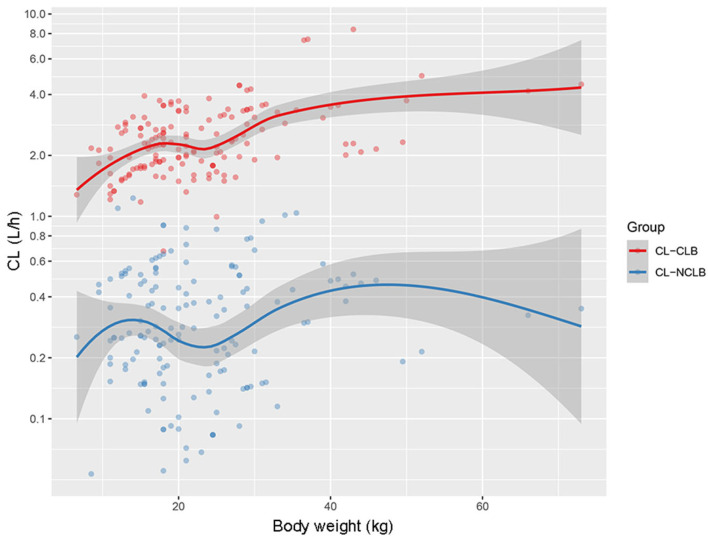
Changes in the clearance of CLB and N-CLB with body weight.

**Figure 4:**
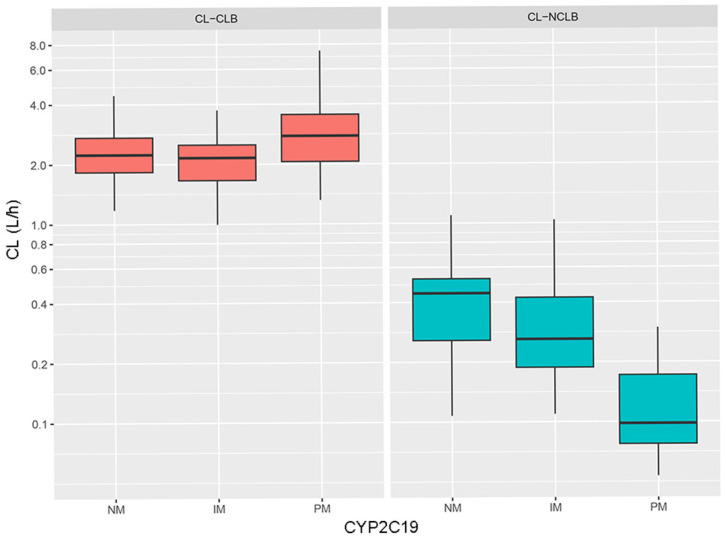
The clearance of CLB and N-CLB in CYP2C19 NMs, IMs, and PMs. Each box-and-whisker plot depicts the distribution of CL across CYP2C19 genotype groups by showing medians (horizontal line), interquartile ranges (box, 25th–75th percentiles), and upper and lower whiskers (5th–95th percentiles).

The clearance rates of both CLB and N-CLB had a tendency to increase at an allometric rate with body weight, as shown by the locally estimated scatterplot smoothing (LOESS) curve (Figure 3). In the figure, the CL for CLB is higher than that of N-CLB at the same body weight. Changes in the clearance of CLB and N-CLB in the context of different CYP2C19 genotypes are also shown (Figure 4). The mean CL_CLB_/F values were 2.39 L·h^−1^, 2.18 L·h^−1^, and 3.10 L·h^−1^ for the NMs, IMs, and PMs, respectively. There was no significant difference in CL_CLB_/F among the three groups (*p* > 0.05). The mean CL_N-CLB_/F_m_ values were 0.46 L·h^−1^, 0.34 L·h^−1^, and 0.13 L·h^−1^ for the NMs, IMs, and PMs, respectively. The CL_N-CLB_/F_m_ was lower in the PMs than in the NMs and IMs, with statistically significant differences (*p* < 0.01). The median values of CL for CLB were negligibly affected by the CYP2C19 genotype, whereas those of N-CLB were significantly decreased in the CYP2C19 PMs compared to the NMs and IMs.

**Figure 5:**
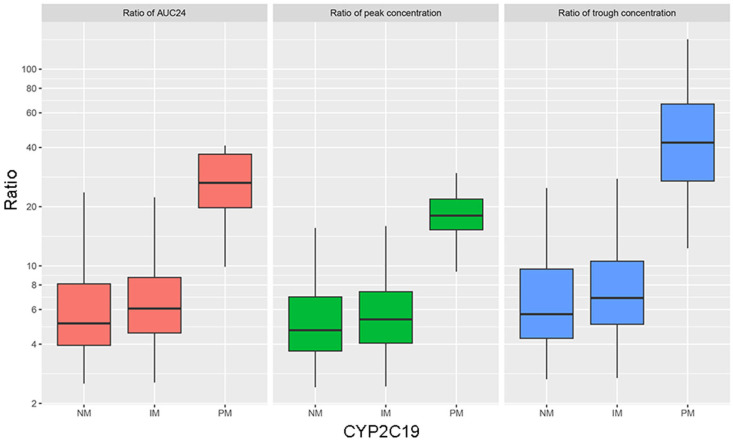
The ratios of N-CLB to CLB in different CYP2C19 genotype groups related to AUC_24h_ (red), C_max_ (green), and C_min_ (blue) in a steady state estimated using the maximum a posteriori Bayesian method. The box displays the interquartile range (25th–75th percentiles), while the whiskers extend to the 5th–95th percentile range. The horizontal line within each box represents the median value.

To elucidate the impact of CYP2C19 pharmacogenetic variability, the ratios of N-CLB to CLB in different CYP2C19 genotype groups related to simulated AUC_24h_, C_max_, and C_min_ values were examined, as depicted in Figure 5. The mean ratios of AUC_24h_ were 6.77, 8.28, and 27.05 for the NMs, IMs, and PMs, respectively; the mean ratios of C_max_ were 5.81, 6.74, and 18.54 for the NMs, IMs, and PMs; and the mean ratios of C_min_ were 8.18, 10.79, and 51.40 for the NMs, IMs, and PMs, respectively. There were no significant differences in the mean ratios of AUC_24h_, C_max_, and C_min_ between the NMs and IMs (*p* > 0.05). However, the mean ratios of AUC_24h_, C_max_, and C_min_ were significantly increased for the PMs compared with the NMs and IMs (*p* < 0.01).

### 3.4. Model-Informed Dosing Regimen

**Figure 6:**
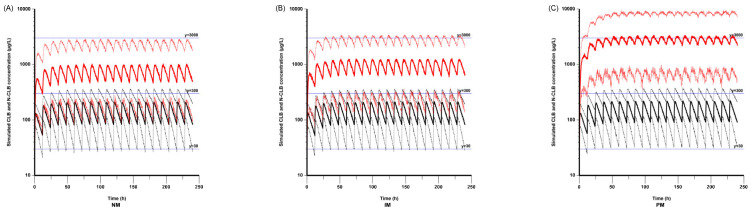
The simulated concentration–time profiles for CLB and N-CLB when a 20 kg subject was treated with CLB at the dose of 0.2 mg·kg^−1^ every 12 h for 10 consecutive days without any cotreatment in CYP2C19 (**A**) NM; (**B**) IM; (**C**) PM. The median values of the simulated CLB and N-CLB concentrations are represented by solid black and red lines, respectively, while their 10th–90th percentile ranges are shown with corresponding dashed lines.

Since body weight and CYP2C19 genotypes were incorporated into the final model, pharmacokinetic simulations were performed for several scenarios to assess clinical practice applications. For a case in which CLB was administrated to a 20 kg subject (the median body weight of the study population) without any cotreatment at a dose of 0.2 mg·kg^−1^ every 12 h for 10 consecutive days, the simulated concentration–time profiles for CLB and N-CLB in the CYP2C19 NMs, IMs, PMs are illustrated in Figure 6. Monte Carlo simulations demonstrate that the steady-state trough concentrations of CLB had no significant difference between the CYP2C19 NMs, IMs, and PMs, and most were within the range of 30–300 μg·L^−1^. On the other hand, the steady-state trough concentrations of N-CLB gradually increased in the order NMs < IMs < PMs. Approximately 90% of the N-CLB concentrations in the NMs and IMs were less than 3000 μg·L^−1^, but about 50% of the N-CLB concentrations in the PMs exceeded 3000 μg·L^−1^.

The simulated plasma concentration profiles and PTA data on different body weights, classified into three groups based on CYP2C19 genotypes, are depicted in Supplementary Tables S4–S6. For the NM and IM children, target therapeutic concentrations of both CLB and N-CLB were achieved by adjusting the dosage, but this outcome was not accomplished in PMs owing to the accumulation of N-CLB in vivo. Considering toxicity and efficacy, the optimal dosing regimens were determined, as shown in Table 3. With this strategy, both PTAs of a C_min_ of CLB ≥ 30 μg·L^−1^ and a C_min_ of N-CLB ≥ 300 μg·L^−1^ were higher than 80% in the NMs and IMs. However, due to the accumulation of N-CLB in the PMs, to ensure safety, the recommended doses in Table 3 could only guarantee that more than 80% of the patients had trough concentrations of N-CLB ≥ 300 μg·L^−1^ and that more than 50% of the patients had trough concentrations of CLB ≥ 30 μg·L^−1^. For laboratory alerts, the PTAs of C_min_ of CLB ≥ 500 μg·L^−1^ recommended in Table 3 were less than 0.1%, but the PTAs of C_min_ of N-CLB ≥ 5000 μg·L^−1^ were between 0.1% (NMs and IMs) and 2.6% (PMs).

## 4. Discussion

In this study, a PPK model of CLB and N-CLB children with epilepsy was developed based on real-world data. A one-compartment model with first-order elimination provided a comprehensive description of the influence of body weight and CYP2C19 genotypes on the pharmacokinetic characteristics of CLB and N-CLB. The major finding of this study was that the current CLB dose might increase the risk of adverse reactions, especially in patients who are CYP2C19 PMs.

Given ethical constraints, an opportunistic sampling strategy was adopted as the primary study design in this investigation. Concurrently, the absence of prior pharmacokinetic models based on rich data prevented sampling strategy optimization. Owing to the limited pharmacokinetic sampling during the absorption and distribution phases, we developed a simplified pharmacokinetic model by reducing model compartments, freezing parameter values, and ignoring interindividual variability for certain parameters. Although the simplified one-compartment model with first-order elimination could not fully characterize the absorption and distribution kinetics of CLB and N-CLB in the pediatric patients, validation analyses (nonparametric bootstrap analysis, GOF, VPCs, and pc-VPCs) demonstrated that the one-compartment model with first-order elimination confirmed its adequate predictive performance. Furthermore, the tandem one-compartment model used in this study has been well-documented in previous PPK studies of CLB in both pediatric and adult populations [19,20]. The pharmacokinetic parameter values in our study presented some differences from previous population studies in children. The estimates of CL/F and V/F corrected for an adult standard body weight of 70 kg for CLB were 5.66 L·h^−1^ and 92.07 L in our study, both of which are higher than those described in Jullien et al., which were 1.23 L·h^−1^ and 39.1 L, respectively [19]. A likely explanation for this discrepancy could be race, comedication, and sample size. Saruwatari et al. reported estimates of CL/F and V/F for CLB of 0.511 L·h^−1^ and 13.3 L, respectively, in Japanese patients with epilepsy [20]. Indeed, a bioavailability correction was applied in the Japanese study, while this study used a CLB-to-N-CLB conversion factor, preventing a parallel comparison because of the difference in correction factors for clearance. However, our results are consistent with earlier studies that found that for single and multiple oral doses of CLB up to 40 mg per day, the average apparent volume of distribution for CLB in a steady state ranged from 99 L to 120 L, indicating that CLB is distributed to peripheral tissues [25]. Therefore, the difference in pharmacokinetic parameters between our population and other populations may need further investigation.

Previous PPK models incorporated age and body weight, which have a well-established association with CLB and N-CLB CL, as covariates. In our model, body weight was found to affect the CL of both CLB and N-CLB, similar to the conclusions of Jullien et al. and Tolbert et al. [19,21]. However, no significant differences in CLB and N-CLB CLs were found regarding age in our model, a result that disagrees with a previous study [21]. This disparity may be attributable to populations of different ages. Our model was conducted in a pediatric population (aged 0.85–16.75 years) rather than a combined pediatric and adult population (aged 6 months to 45 years), thus preventing meaningful comparisons. Additional research is necessary to explore the effect of age on CLB and N-CLB exposure in children.

The ketogenic diet and comedication remain the most commonly used strategies for children with epilepsy undergoing CLB treatment. The ketogenic diet, which is used as add-on therapy in epilepsy, was not included in our final model because related data did not satisfy the criteria in our stepwise process. However, a case study verified that when a patient started a ketogenic diet concurrent with CLB treatment, there was a 42% decrease in CLB and N-CLB serum concentrations [26]. A lack of samples made it difficult to draw any conclusions about the effect of the ketogenic diet on CLB pharmacokinetics. Comedication is a crucial factor in improving antiepileptic outcomes when CLB is applied. Our study demonstrated that concomitantly used antiepileptic drugs were not influential covariates for CLB and N-CLB exposure. Similar to previous studies, there were no clinically relevant drug–drug interactions with selected antiepileptic drugs, based on a pharmacokinetic modeling approach with grouped comedication [27,28]. Although many antiepileptic drugs have been reported to affect the activity of CYP3A4 and CYP2C19, such as valproic acid and oxcarbazepine, in this study, these drugs demonstrated no effects on the pharmacokinetics of CLB and N-CLB, which was also found by another study [27]. More samples are required to continue this study.

Among the potential organs related to drug disposal, the liver and kidneys have attracted special attention. A study by Tolbert et al. demonstrated that liver impairment had no significant effect on the clearance of CLB and N-CLB [29]. Similarly, our results confirm the previous report. For patients with mild or moderate renal insufficiency, CLB/N-CLB changes in blood concentration or renal clearance were not clinically significant. Patients with renal insufficiency do not need their dose to be adjusted. Since there is no evidence that renal function is related to any pharmacokinetic parameters of CLB or N-CLB after single or multiple dosing, no dose adjustment is necessary for patients with mild or moderate renal disease. Our study yielded similar results. As the number of patients with impaired kidney function was limited, it was not possible to draw any firm conclusions regarding the impact of kidney function on the pharmacokinetic profiles of CLB and N-CLB.

Genetic polymorphisms of drug-metabolizing enzymes have been assumed to be responsible for the interindividual variability in CLB pharmacokinetics; however, no clinical guidelines or recommendations based on genetically determined metabolism have been prepared for CLB dosing in Chinese epilepsy patients. Many researchers have examined the SNPs responsible for the metabolism and clearance of CLB and N-CLB. These include genes involved in ABCB1, CYP3A4, and CYP2C19. In our study, SNPs in ABCB1 and CYP3A4 did not show a statistically significant effect on CLB and/or N-CLB pharmacokinetics, whereas CYP2C19 had an important influence on N-CLB clearance. CYP2C19 is the primary enzyme involved in the metabolism of N-CLB to 4′-hydroxy-n-demethylclobazole; the CYP2C19 genotype (NM, IM, or PM) had a greater impact on the clearance of N-CLB (0.46 ± 0.20, 0.34 ± 0.23, 0.13 ± 0.08, respectively) than on the clearance of CLB (2.39 ± 0.77, 2.18 ± 0.70, 3.10 ± 1.54, respectively), indicating a gene–dose effect. The clearance of N-CLB was significantly lower, i.e., 71.7% lower, in PMs compared with NMs, which is slightly lower than the 84.9% reported by Saruwatari et al. [20]. Thus, our study confirms the reduced metabolism ability of PMs compared to NMs and IMs, which is in accordance with previous studies [20,30]. Furthermore, the study cohort included 41 (39.81%), 45 (43.69%), and 15 (14.56%) subjects with CYP2C19 NM, IM, and PM genotypes, respectively. Stepwise covariate analysis (Supplementary Table S3) identified the CYP2C19 genotype as a statistically significant covariate influencing N-CLB clearance. The RSE (%) values for the final model parameters (θ_CYP2C19,IM_ and θ_CYP2C19,PM_) were below 50%, with less than 5% bias between the bootstrap median and final model estimates. These results confirm the model’s robustness and demonstrate adequate statistical power to characterize the variability in N-CLB clearance mediated by CYP2C19.

The N-CLB/CLB ratio has been proposed as a tool for assessing the CYP2C19 activity phenotype. In this study, we showed that the CYP2C19 PMs presented with four times, three times, and six times higher N-CLB/CLB ratios of AUC_24h_, C_max_, and C_min_, respectively, compared with the values observed for the NMs. In addition, our study found that the N-CLB/CLB ratios in the IMs were higher than those in the NMs, which is concomitant with the findings of Giraud et al. [13]. In a previous study, N-CLB/CLB ratios were 10- to 27-fold higher in CYP2C19 PMs than the median values of the control epileptic patients, indicating a higher risk of adverse effects in PMs [31]. Although the increase in our ratios was lower than that in the previous report, both studies show similar trends and suggest poor clearance of N-CLB in PMs.

According to the simulated concentrations in the NMs, IMs, and PMs based on a patient mass of 20 kg, the N-CLB concentrations were higher in the PMs compared with the NMs and IMs, while the CLB concentrations were not significantly different among the three CYP2C19 genotype groups. This result aligns with that of a previous study [30]. It has been reported that the mean N-CLB concentrations in PMs were 8.4 and 3.6 times higher than those in NMs and IMs, respectively [32,33]. Although our results did not involve a numerical comparison, higher concentrations of N-CLB in the PM population were also observed in our study. An extensive literature review revealed no previously established pediatric-specific target exposure ranges or alert thresholds for CLB and N-CLB. Pediatric–adult pharmacodynamic disparities in receptor affinity and biological responses may necessitate distinct therapeutic exposure targets. However, current clinical practice typically applies identical target exposure ranges and safety thresholds for both pediatric and adult populations, consistent with established guidelines [19]. This study combined actual measurements with Bayesian estimation to obtain the following results. Under a real-world pediatric dosing regimen (twice daily if >5 mg; starting dose of 5 mg for ≤30 kg, 10 mg for >30 kg, with subsequent individual adjustments based on efficacy and tolerability) with therapeutic drug monitoring-guided adjustments, 85.4% (88/103) of the patients achieved therapeutic CLB trough concentrations of 30–300 μg·L^−1^, and 79.6% (82/103) attained the target N-CLB trough concentrations of NCLB 300–3000 μg·L^−1^. These findings are consistent with the existing adult-derived therapeutic targets, with no treatment-limiting adverse events observed. However, due to the absence of a rigorous experimental design, the exposure–response relationship could not be definitively established. Therefore, based on the observed drug concentration distribution and safety correlation data, this study adopted simulated target concentration ranges of 30–300 μg·L^−1^ for CLB and 300–3000 μg·L^−1^ for N-CLB, consistent with the established therapeutic ranges reported in the previous literature [17,19]. For safety considerations, alert thresholds for both CLB and N-CLB were maintained at established adult reference levels, given the limited evidence from cases exceeding these thresholds in pediatric populations, despite potential age-related differences in optimal thresholds. In addition, the N-CLB concentrations in the CYP2C19 PM population exceeded the upper therapeutic range (3000 μg·L^−1^) in our simulation, implying a greater possibility of adverse effects in this population during the therapeutic period. This also means that the current dose strategy might result in over-dosing in PMs, and a lower dose of CLB should be considered for these patients.

In this study, we also simulated steady-state CLB and N-CLB trough concentrations based on different CYP2C19 genotypes in subjects weighing 10 kg to 50 kg. As a result, the plasma concentrations of N-CLB were higher in the CYP2C19 PMs than in those of NMs and IMs, even when a lower dose of CLB was given at the same body weight. It has been reported that the seizure reduction rate is associated with the concentration of N-CLB rather than the concentration of CLB, associating higher N-CLB concentrations with better seizure control [30]. Due to difficulty in evaluating the efficacy of CLB in children with epilepsy, our study did not examine pharmacodynamics.

Ideally, CLB therapy should be initiated at a low dose (2.5 mg per day), and the dosage should be increased until the N-CLB serum concentration reaches 1100 μg·L^−1^ or the desired effect is acquired, which is particularly important for PMs [30]. A PPK study indicates that effective blood concentrations of CLB and N-CLB can be achieved in pediatric patients as young as 16 months old when dosed with 1.5 and 2.0 mg·kg^−1^ of CLB; however, adverse effects should be monitored when doses exceed 1.5 mg·kg^−1^, especially in patients under 2 years old [21]. Another PPK study suggests that doses of CLB at 1.0 to 2.0 mg·kg^−1^ per day are beneficial for patients with clinically refractory epilepsy [34]. Our simulation results show that CLB treatment at a dose of 0.1 mg·kg^−1^ twice daily in PMs with a body weight of 10 kg can achieve ideal trough concentrations, while doses higher than 0.1 mg·kg^−1^ twice daily are needed to achieve efficient concentrations in NMs and IMs. Therefore, monitoring plasma CLB and N-CLB concentrations and determining the CYP2C19 genotype can be effective tools for individualized treatment of CLB.

Our integrated analysis combining empirical measurements with Bayesian estimation revealed that the mean steady-state trough concentration of N-CLB was 51.4-fold higher than that of CLB in the CYP2C19 PMs (Figure 5). Simulation studies further confirmed significant accumulation of N-CLB in PMs. As a result, conventional dosing regimens failed to achieve therapeutic targets for both compounds simultaneously. Combining experimental observations and modeling results, this study identified metabolic differences between CLB and N-CLB in PMs and assessed the potential for toxic risk generation. Given the paramount importance of patient safety in clinical practice, we prioritized N-CLB concentration for dose optimization. The current dosing regimen might be inadequate to achieve CLB’s full therapeutic effect. While this dosing strategy maintained N-CLB concentrations above alert thresholds in <3% of the cases, only approximately 50% of the patients achieved therapeutic CLB trough concentrations (≥30 μg·L^−1^). In the CYP2C19 PMs, this created an important clinical trade-off, as relying exclusively on N-CLB as a dose-adjustment biomarker could result in subtherapeutic CLB levels. This study suggests that for PM patients, the N-CLB concentration should serve as the primary biomarker for dose adjustment. If seizure control remains inadequate, a protocol of gradual dose escalation should be initiated, accompanied by comprehensive therapeutic drug monitoring (including both CLB and N-CLB levels) and rigorous adverse event monitoring. Ultimately, an individualized dosage can be established to optimally balance efficacy and safety.

This study has some limitations. First, the opportunistic sampling scheme led to suboptimal data point distribution in pediatric patients, compromising the model’s precision in characterizing both the pharmacokinetic processes (absorption/distribution) and the biotransformation of CLB to N-CLB. Second, the model was not externally validated and therefore cannot be applied to other groups. Based on the GOF and pc-VPC plots, the current model demonstrates suboptimal predictive accuracy for both high- and low-concentration observations. Third, since the available data unfortunately lack UM and RM patients, the impact of SNPs on CLB and N-CLB pharmacokinetics may not be fully assessed. This restricts the model’s utility in populations where these phenotypes are predominant (e.g., American, European, and Latino cohorts), necessitating future validation. Therefore, a larger dataset is necessary for future studies.

## 5. Conclusions

This study is the first to comprehensively evaluate the effects of developmental and genetic factors on the pharmacokinetics of CLB in Chinese children with epilepsy and to propose optimized dosing regimens based on the model. The results indicate that the usual twice-daily 0.2 mg·kg^−1^ dose of CLB is not appropriate for pediatric patients with different genetic backgrounds. Dosing regimens stratified by body weight and CYP2C19 would improve the probability of achieving therapeutic exposure levels for both CLB and N-CLB, resulting in better clinical outcomes.

## Figures and Tables

**Table 1:** A summary of the demographic and clinical characteristics of the enrolled subjects.

	Number	Mean (SD)	Median (Range)
Patients	103		
Gender (M:F)	58:45		
Age (years)		5.94 (3.15)	5.46 (0.85–16.75)
Weight (kg)		22.94 (10.48)	20.00 (6.60–73.00)
Height (cm)		113.62 (19.57)	112.00 (71.00–171.00)
Body surface area (m^2^)		0.84 (0.25)	0.80 (0.36–1.81)
Blood urea nitrogen (mmol·L^−1^)		4.75 (2.69)	4.45 (0.60–31.80)
Serum creatinine concentration (μmol·L^−1^)		34.29 (12.43)	32.95 (12.80–133.80)
Uric acid (μmol·L^−1^)		249.89 (81.85)	241.00 (72.00–606.00)
Cystatin C (mg·L^−1^)		0.84 (0.13)	0.84 (0.57–1.34)
Estimated glomerular filtration rate (mL·min^−1^·(1.73 m^2^)^−1^)		128.56 (29.36)	126.17 (33.33–240.85)
Alanine aminotransferase (U·L^−1^)		14.51 (10.94)	12.00 (1.00–72.00)
Aspartate aminotransferase (U·L^−1^)		29.59 (12.58)	26.00 (11.00–94.00)
Total protein (g·L^−1^)		63.68 (6.03)	63.70 (38.50–84.50)
Albumin (g·L^−1^)		43.03 (3.94)	43.70 (30.00–51.90)
Globulin (g·L^−1^)		20.65 (4.94)	20.00 (8.40–43.20)
Albumin/globulin ratio		2.20 (0.53)	2.20 (0.90–3.80)
Total bilirubin (μmol·L^−1^)		4.97 (2.39)	4.70 (1.50–16.80)
Direct bilirubin (μmol·L^−1^)		2.26 (1.33)	2.00 (0.10–12.10)
Alkaline phosphatase (U·L^−1^)		230.35 (77.96)	228.00 (53.00–656.00)
γ-glutamyl transpeptidase (U·L^−1^)		30.33 (32.15)	20.00 (5.00–274.00)
Prealbumin (mg·L^−1^)		200.26 (46.03)	199.80 (20.70–410.00)
CLB concentration (μg·mL^−1^)		137.01 (92.74)	114.50 (15.40–495.00)
N-CLB concentration (μg·mL^−1^)		1611.56 (1385.85)	1115.00 (55.90–7060.00)
CYP2C19 genotypes			
RMs	2 (1.94%)		
NMs	41 (39.81%)		
IMs	45 (43.69%)		
PMs	15 (14.56%)		

SD: standard deviation; M:F, male–female.

**Table 2:** Pharmacokinetic parameter estimates from the final model and bootstrap evaluations.

Parameter	Final Model		Bootstrap Result		Bias (%)
	Estimate	RSE (%)	Median	95%CI	
Ka (h^−1^)	1.99 (fixed)	—	1.99 (fixed)	—	—
V_CLB_/F (L)	92.07	31.15	93.08	40.42–176.27	1.10
CL_CLB_/F (L^−1^)	5.66	10.53	5.63	4.09–7.26	−0.53
V_N-CLB_/F_m_ (L)	1.84	29.66	1.87	0.72–2.76	1.63
CL_N-CLB_/F_m_ (L^−1^)	1.01	11.83	1.01	0.71–1.30	0
θ_CYP2C19,NM_	0.00 (fixed)	—	0.00 (fixed)	—	—
θ_CYP2C19,IM_	−0.25	39.03	−0.24	−0.53–−0.02	4.00
θ_CYP2C19,PM_	−1.30	15.01	−1.28	−1.67–−0.89	1.54
Interindividual variation					
ω^2^CL_CLB/F_ (%)	15.94	25.85	15.97	7.31–24.63	0.19
ω^2^_CLN-CLB_/_Fm_ (%)	45.35	20.20	44.96	25.28–64.64	−0.86
η-Shrinkage-CL_CLB_ (%)	9.73	—	—	—	—
η-Shrinkage-CL_N-CLB_ (%)	17.42	—	—	—	—
Intraindividual variation					
σ_CLB_	0.33	8.52	0.33	0.27–0.37	0
σ_N-CLB_	0.53	8.60	0.53	0.44–0.61	0
ε-Shrinkage (%)	33.17	—	—	—	—

RSE, relative standard error; CI, confidence interval; Ka, absorption rate constant of clobazam; V_CLB_/F, volume of distribution of clobazam; CL/F, clearance of clobazam; V_N-CLB_/F_m_, volume of distribution of N-desmethylclobazam; CL_N-CLB_/F_m_, clearance of N-desmethylclobazam; θ_CYP2C19_, the fixed effect values of CL_N-CLB_/F_m_ affected by different genotypes; ω^2^CL_CLB_/F, ω^2^CL_N-CLB_/F_m_, interindividual variance for pharmacokinetic parameters; σ, residual variability; —: not applicable (N/A).

**Table 3:** Optimal simulated dose strategies based on body weight and CYP2C19 genotypes and corresponding PTA (%).

CYP2C19 Genotype	Body Weight (kg)	Dosage (mg/kg, Twice Daily)	PTA (%) of CLB Trough Concentration				PTA (%) of N-CLB Trough Concentration			
			Median Concentration (μg·L^−1^)	≥30 μg·L^−1^	≥300 μg·L^−1^	≥500 μg·L^−1^	Median Concentration (μg·L^−1^)	≥300 μg·L^−1^	≥3000 μg·L^−1^	≥5000 μg·L^−1^
NM	10	0.3	109.28	94.8	4.2	0	629.52	84.1	2.5	0.3
	20	0.25	119.74	97.0	5.1	0.1	699.26	88.4	2.7	0.4
	30	0.2	111.47	97.0	3.5	0	651.02	86.3	2.2	0.2
	40	0.175	108.24	97.2	2.5	0	630.81	85.5	2.0	0.1
	50	0.15	93.03	95.6	0.9	0	568.99	80.9	1.7	0.2
IM	10	0.25	83.75	89.6	1.4	0	658.07	83.5	4.3	1.2
	20	0.2	88.17	93.5	1.3	0	708.52	87.1	3.8	0.6
	30	0.15	77.19	92.1	0.6	0	626.37	82.7	2.6	0.2
	40	0.125	71.53	91.7	0.3	0	579.29	80.9	1.8	0.2
	50	0.12	80.33	94.0	0.2	0	616.59	84.4	1.6	0.1
PM	10	0.1	33.50	57.3	0	0	962.78	89.9	9.3	2.6
	20	0.075	33.06	55.9	0	0	950.00	92.2	6.3	1.1
	30	0.075	38.59	66.1	0	0	1081.81	95.2	7.7	2.0
	40	0.0625	35.94	62.3	0	0	967.24	91.4	7.2	1.7
	50	0.06	37.39	65.2	0	0	996.10	92.0	7.7	1.9

## Data Availability

The data that support the findings of this study are available from the corresponding authors upon reasonable request. The data are not publicly available due to ethical reasons as per local guidelines.

## Supplementary Materials

The following supporting information can be downloaded at https://www.mdpi.com/article/10.3390/pharmaceutics17070813/s1, Figure S1: The OFVs affected by comedication; Figure S2: The OFVs affected by the SNPs of ABCB1, CYP3A4, and GABA receptors; Table S1: Genotype frequencies of ABCB1, CYP3A4, and GABA receptors; Table S2: The frequency of co-medicated drugs and the ketogenic diet in this study; Table S3: The OFV of the basic and covariate models used in our PPK analysis; Table S4: Simulated dose strategy based on body weight and CYP2C19 NMs and corresponding PTA (%); Table S5: Simulated dose strategy based on body weight and CYP2C19 IMs and corresponding PTA (%); Table S6: Simulated dose strategy based on body weight and CYP2C19 PMs and corresponding PTA (%).
