# Supplementary material for: Population Pharmacokinetics and Model-Informed Precision Dosing of Clobazam Based on the Developmental and Genetic Characteristics of Children with Epilepsy
Source: Pharmaceutics. 2025 Jun 23;17(7):813. doi: 10.3390/pharmaceutics17070813 (PMC12300161; doi:10.3390/pharmaceutics17070813)
Supplement: Supplementary file 1 [file pharmaceutics-17-00813-s001.zip › Supplementary table/Supplemental table 1.pdf]

Supplemental table S1. Genotype frequencies of ABCB1, CYP3A4, and GABA receptors.

| SNP (rsID) | Genotype | Number (n) | Frequency (%) |
|---|---|---|---|
| ABCB1 | | | |
| rs1128503 | CC/CT/TT | 17/51/35 | 16.50%/49.51%/33.98% |
| rs2032582 | GG/TT/AA/GT/TA/GA | 14/32/22/15/4/16 | 13.59%/31.07%/21.36%/14.56%/3.88%/15.53% |
| rs1045642 | CC/CT/TT | 44/49/10 | 42.72%/47.57%/9.71% |
| CYP3A4*1B | | | |
| rs2740574 | TT | 103 | 100% |
| CYP3A4*1G | | | |
| rs2242480 | TT/TC/CC | 5/41/56 | 4.90%/40.20%/54.90% |
| GABRA1 | | | |
| rs2279020 | AA/AG/GG | 13/54/36 | 12.62%/52.43%/34.95% |
| GABRG1 | | | |
| rs279858 | TT/TC/CC | 20/51/32 | 19.42%/49.51%/31.07% |
| GABRA2 | | | |
| rs11503014 | CC/CG | 88/15 | 85.44%/14.56% |
| GABRB2 | | | |
| rs2229944 | GG/AG | 93/10 | 90.29%/9.71% |
| GABRG2 | | | |
| rs211014 | CC/CA/AA | 28/48/27 | 27.18%/46.60%/26.21% |
| rs211037 | CC/CT/TT | 15/51/37 | 14.56%/49.51%/35.92% |
