# Supplementary material for: Population Pharmacokinetics and Model-Informed Precision Dosing of Clobazam Based on the Developmental and Genetic Characteristics of Children with Epilepsy
Source: Pharmaceutics. 2025 Jun 23;17(7):813. doi: 10.3390/pharmaceutics17070813 (PMC12300161; doi:10.3390/pharmaceutics17070813)
Supplement: Supplementary file 1 [file pharmaceutics-17-00813-s001.zip › Supplementary table/Supplemental table 2.pdf]

Supplemental table S2. The frequency of co-medicated drugs and ketogenic diet in this study.

Co-medicated drugs	Number (n)	Frequency
Valproic acid	83	80.58%
Lamotrigine	29	28.16%
Perampanel	25	24.27%
Levetiracetam	19	18.45%
Topiramate	18	17.48%
Zonisamide	14	13.59%
Lacosamide	11	10.68%
Oxcarbazepine	11	10.68%
Clonazepam	4	3.88%
Vigabatrin	3	2.91%
Rufinamide	2	1.94%
Stiripentol	2	1.94%
Nitrazepam	1	0.97%
Ketogenic diet	7	6.80%
