# Supplementary material for: Population Pharmacokinetics and Model-Informed Precision Dosing of Clobazam Based on the Developmental and Genetic Characteristics of Children with Epilepsy
Source: Pharmaceutics. 2025 Jun 23;17(7):813. doi: 10.3390/pharmaceutics17070813 (PMC12300161; doi:10.3390/pharmaceutics17070813)
Supplement: Supplementary file 1 [file pharmaceutics-17-00813-s001.zip › Supplementary table/Supplemental Table 3.pdf]

Supplemental table S3. The OFV of basic and covariate models used in our PPK analysis.

Step	Model name/Stracture	OFV	△OFV	P value
0	Basic model	4206.28	–	–
Forward inclusion				
1	Allometric model	4146.34	– 59.94	–
2	Allometric model+$CL_{N\text{-}CLB}$-CYP2C19	4119.90	– 26.44	< 0.001
3	Allometric model+$CL_{N\text{-}CLB}$-CYP2C19+ $CL_{N\text{-}CLB}$-AST	4112.62	– 7.28	< 0.01
Backward elimination				
4	Allometric model+ $CL_{N\text{-}CLB}$-CYP2C19	4119.90	7.28	> 0.001
