# Supplementary material for: Population Pharmacokinetics and Model-Informed Precision Dosing of Clobazam Based on the Developmental and Genetic Characteristics of Children with Epilepsy
Source: Pharmaceutics. 2025 Jun 23;17(7):813. doi: 10.3390/pharmaceutics17070813 (PMC12300161; doi:10.3390/pharmaceutics17070813)
Supplement: Supplementary file 1 [file pharmaceutics-17-00813-s001.zip › Supplementary table/Supplemental Table 4 CYP2C19 NM.pdf]

Supplemental table S4. Simulated dose strategy based on body weight in CYP2C19 NMs and corresponding PTA (%).

Group	Dosage	PTA (%) of CLB trough concentration				PTA (%) of N-CLB trough concentration			
		Median concentration ($\mu g{\cdot}L^{-1}$)	≥30 $\mu g{\cdot}L^{-1}$	≥300 $\mu g{\cdot}L^{-1}$	≥500 $\mu g{\cdot}L^{-1}$	Median concentration ($\mu g{\cdot}L^{-1}$)	≥300 $\mu g{\cdot}L^{-1}$	≥3000 $\mu g{\cdot}L^{-1}$	≥5000 $\mu g{\cdot}L^{-1}$
10 kg	0.1 mg/kg, twice daily	33.50	57.3	0	0	199.58	33.4	0.1	0
	0.2 mg/kg, twice daily	66.63	86.4	0.5	0	396.69	63.7	0.7	0.1
	0.3 mg/kg, twice daily	109.28	94.8	4.2	0	629.52	84.1	2.5	0.3
20 kg	0.1 mg/kg, twice daily	44.32	71.4	0	0	263.58	43.8	0.3	0
	0.2 mg/kg, twice daily	88.17	93.5	1.3	0	535.39	78.2	1.9	0.2
	0.25 mg/kg, twice daily	119.74	97.0	5.1	0.1	699.26	88.4	2.7	0.4
30 kg	0.1 mg/kg, twice daily	51.72	79.4	0	0	307.11	51.3	0.8	0
	0.2 mg/kg, twice daily	111.47	97.0	3.5	0	651.02	86.3	2.2	0.2
	0.3 mg/kg, twice daily	167.20	98.6	14.5	2.0	976.53	94.8	8.8	1.4
40 kg	0.1 mg/kg, twice daily	57.50	83.8	0	0	345.68	57.3	0,8	0
	0.15 mg/kg, twice daily	92.78	95.3	0.7	0	540.69	79.1	0.8	0
	0.175 mg/kg, twice daily	108.24	97.2	2.5	0	630.81	85.5	2.0	0.1
50 kg	0.1 mg/kg, twice daily	62.32	87.6	0	0	375.56	61.4	0.8	0.1
	0.15 mg/kg, twice daily	93.03	95.6	0.9	0	568.99	80.9	1.7	0.2
	0.175 mg/kg, twice daily	117.15	97.7	3.5	0	681.99	88.8	2.5	0.2
