# Supplementary material for: Population Pharmacokinetics and Model-Informed Precision Dosing of Clobazam Based on the Developmental and Genetic Characteristics of Children with Epilepsy
Source: Pharmaceutics. 2025 Jun 23;17(7):813. doi: 10.3390/pharmaceutics17070813 (PMC12300161; doi:10.3390/pharmaceutics17070813)
Supplement: Supplementary file 1 [file pharmaceutics-17-00813-s001.zip › Supplementary table/Supplemental Table 5 CYP2C19 IM.pdf]

Supplemental table S5. Simulated dose strategy based on body weight in CYP2C19 IMs and corresponding PTA (%).

Group	Dosage	PTA (%) of CLB trough concentration				PTA (%) of N-CLB trough concentration			
		Median concentration (μg·L^{-1})	≥30 μg·L^{-1}	≥300 μg·L^{-1}	≥500 μg·L^{-1}	Median concentration (μg·L^{-1})	≥300 μg·L^{-1}	≥3000 μg·L^{-1}	≥5000 μg·L^{-1}
10 kg	0.1 mg/kg, twice daily	33.50	57.3	0	0	263.23	44.2	0.6	0
	0.2 mg/kg, twice daily	66.63	86.4	0.5	0	532.47	77.2	2.0	0.2
	0.25 mg/kg, twice daily	83.75	89.6	1.4	0	658.07	83.5	4.3	1.2
20 kg	0.1 mg/kg, twice daily	44.32	71.4	0	0	349.34	57.7	0.8	0.1
	0.2 mg/kg, twice daily	88.17	93.5	1.3	0	708.52	87.1	3.8	0.6
	0.25 mg/kg, twice daily	119.74	97.0	5.1	0.1	922.97	93.7	8.1	1.2
30 kg	0.1 mg/kg, twice daily	51.72	79.3	0	0	411.90	65.5	0.9	0.2
	0.15 mg/kg, twice daily	77.19	92.1	0.6	0	626.37	82.7	2.6	0.2
	0.2 mg/kg, twice daily	111.47	97.0	3.5	2.0	857.16	93.1	5.8	0.7
40 kg	0.1 mg/kg, twice daily	57.50	83.8	0	0	458.34	70.2	1.2	0.2
	0.125 mg/kg, twice daily	71.53	91.7	0.3	0	579.29	80.9	1.8	0.2
	0.15 mg/kg, twice daily	92.78	95.3	0.7	0	711.92	89.5	2.6	0.3
50 kg	0.08 mg/kg, twice daily	49.86	79.0	0	0	396.67	64.0	0.9	0.1
	0.1 mg/kg, twice daily	62.02	88.4	0.1	0	502.24	74.9	0.9	0.1
	0.12 mg/kg, twice daily	80.33	94.0	0.2	0	616.59	84.4	1.6	0.1
