# Supplementary material for: Population Pharmacokinetics and Model-Informed Precision Dosing of Clobazam Based on the Developmental and Genetic Characteristics of Children with Epilepsy
Source: Pharmaceutics. 2025 Jun 23;17(7):813. doi: 10.3390/pharmaceutics17070813 (PMC12300161; doi:10.3390/pharmaceutics17070813)
Supplement: Supplementary file 1 [file pharmaceutics-17-00813-s001.zip › Supplementary table/Supplemental Table 6 CYP2C19 PM.pdf]

Supplemental table S6. Simulated dose strategy based on body weight in CYP2C19 PMs and corresponding PTA (%).

Group	Dosage	PTA (%) of CLB trough concentration				PTA (%) of N-CLB trough concentration			
		Median concentration (μg·L^{-1})	≥30 μg·L^{-1}	≥300 μg·L^{-1}	≥500 μg·L^{-1}	Median concentration (μg·L^{-1})	≥300 μg·L^{-1}	≥3000 μg·L^{-1}	≥5000 μg·L^{-1}
10 kg	0.1 mg/kg, twice daily	33.50	57.3	0	0	962.78	89.9	9.3	2.6
	0.15 mg/kg, twice daily	49.97	76.4	0	0	1471.78	97.4	17.9	6.0
	0.2 mg/kg, twice daily	72.86	88.6	0.5	0	2021.07	99.0	31.9	13.4
	0.4 mg/kg, once daily	34.75	57.1	0	0	1070.77	89.3	16.8	7.0
20 kg	0.05 mg/kg, twice daily	22.16	30.7	0	0	616.83	80.7	2.7	0.8
	0.075 mg/kg, twice daily	33.06	55.9	0	0	950.00	92.2	6.3	1.1
	0.1 mg/kg, twice daily	47.90	77.5	0	0	1283.64	97.2	13.8	2.7
	0.2 mg/kg, once daily	23.37	40.3	0	0	739.10	81.7	8.6	2.6
30 kg	0.05 mg/kg, twice daily	25.86	41.4	0	0	705.75	85.3	3.4	0.8
	0.075 mg/kg, twice daily	38.59	66.1	0	0	1081.83	95.2	7.7	2.0
	0.1 mg/kg, twice daily	55.73	84.3	0	0	1445.80	98.5	17.4	4.6
	0.2 mg/kg, once daily	29.31	49.1	0	0	915.69	86.6	12.2	3.9
40 kg	0.025 mg/kg, twice daily	14.38	10.2	0	0	386.89	63.4	0.8	0
	0.05 mg/kg, twice daily	28.61	46.9	0	0	788.78	89.4	3.3	0.4
	0.075 mg/kg, twice daily	46.39	77.5	0	0	1196.45	97.1	11.4	2.2
	0.0625 mg/kg, twice daily	35.94	62.3	0	0	967.24	91.4	7.2	1.7
50 kg	0.025 mg/kg, twice daily	15.58	11.8	0	0	415.04	66.6	0.8	0
	0.04 mg/kg, twice daily	24.81	36.1	0	0	674.91	85.4	2.3	0.2
	0.05 mg/kg, twice daily	33.47	59.5	0	0	856.02	93.1	3.7	0.5
	0.06 mg/kg, twice daily	37.39	65.2	0	0	996.10	92.0	7.7	1.9
