# Supplementary figures and images for: Population Pharmacokinetics and Model-Informed Precision Dosing of Clobazam Based on the Developmental and Genetic Characteristics of Children with Epilepsy
Source: Pharmaceutics. 2025 Jun 23;17(7):813. doi: 10.3390/pharmaceutics17070813 (PMC12300161; doi:10.3390/pharmaceutics17070813)

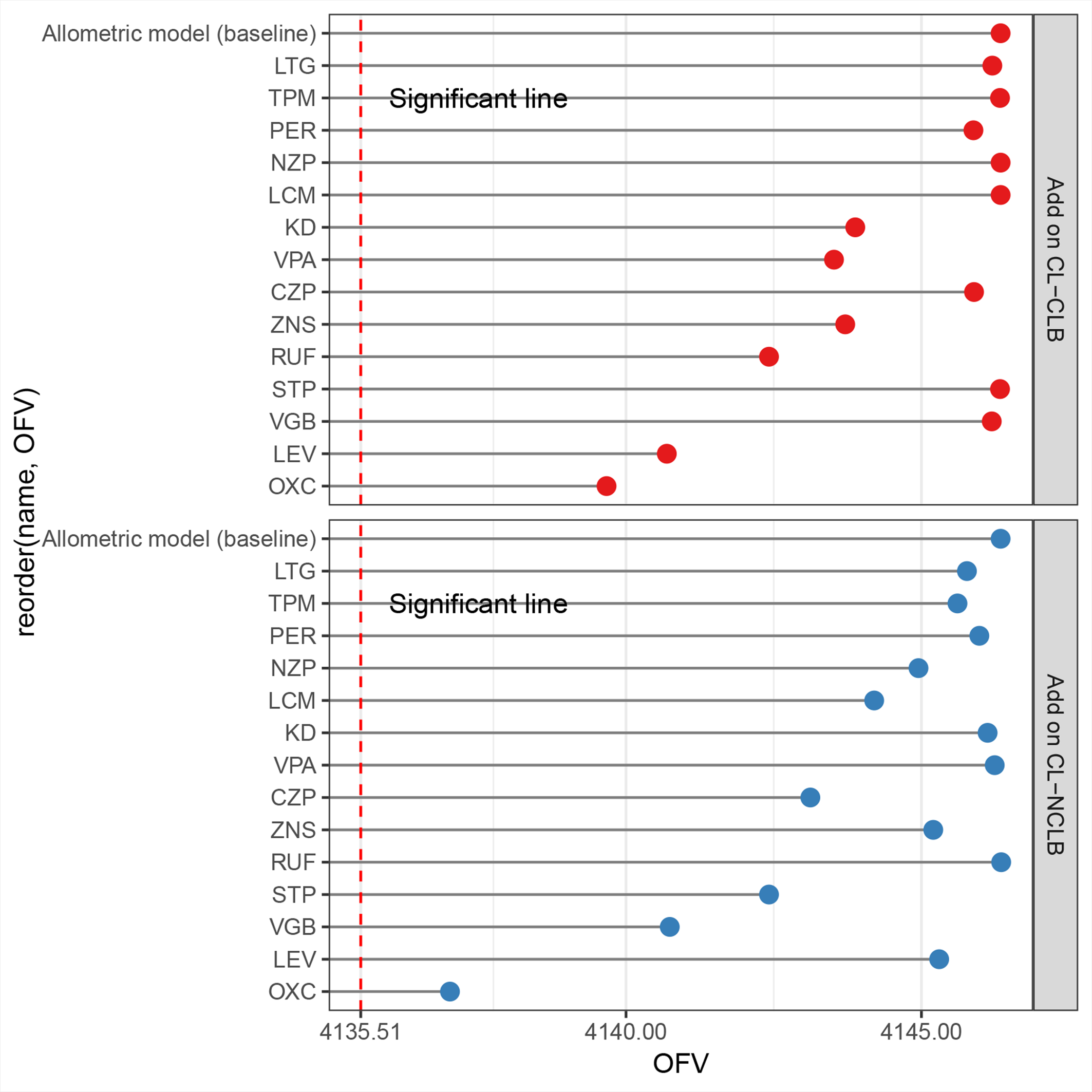

Supplement: Supplementary file 1 [file pharmaceutics-17-00813-s001.zip › Supplementary figure/Supplemental Figure 1.tif]

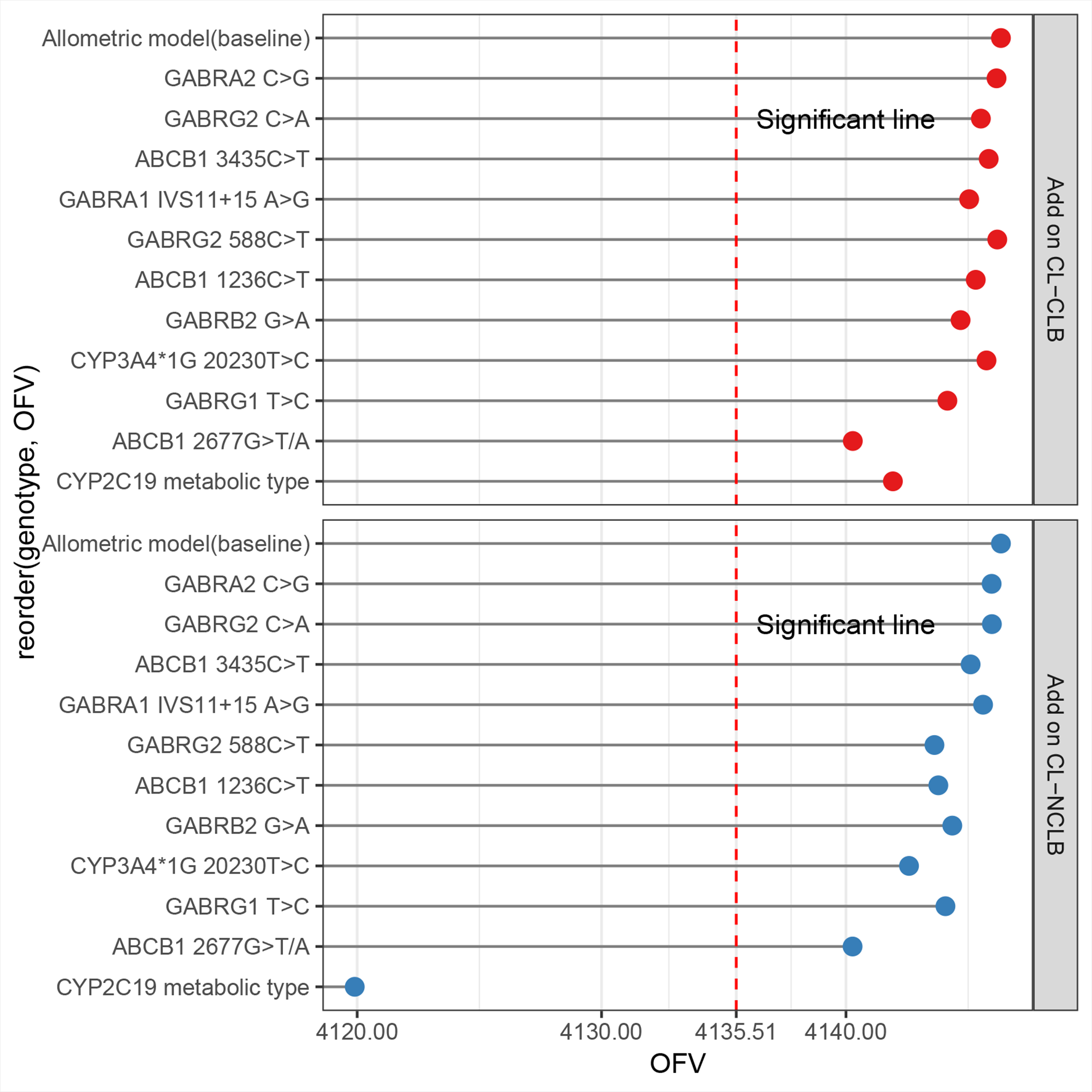

Supplement: Supplementary file 1 [file pharmaceutics-17-00813-s001.zip › Supplementary figure/Supplemental Figure 2.tif]
